# Prolonged mass azithromycin distributions and macrolide resistance determinants among preschool children in Niger: A sub-study of a cluster-randomized trial (MORDOR)

**DOI:** 10.1371/journal.pmed.1004386

**Published:** 2024-05-06

**Authors:** Ahmed M. Arzika, Amza Abdou, Ramatou Maliki, Nassirou Beido, Boubacar Kadri, Abdoul N. Harouna, Abdoul N. Galo, Mankara K. Alio, Elodie Lebas, Catherine E. Oldenburg, Kieran S. O’Brien, Cindi Chen, Lina Zhong, Zhaoxia Zhou, Daisy Yan, Armin Hinterwirth, Jeremy D. Keenan, Travis C. Porco, Thomas M. Lietman, Thuy Doan, for the MORDOR Study Group

**Affiliations:** 1 Programme National de Santé Oculaire, Niamey, Niger; 2 Francis I Proctor Foundation, University of California, San Francisco, California, United States of America; 3 Department of Ophthalmology, University of California, San Francisco, California, United States of America; 4 Department of Epidemiology & Biostatistics, University of California, San Francisco, California, United States of America

## Abstract

**Background:**

Randomized controlled trials found that twice-yearly mass azithromycin administration (MDA) reduces childhood mortality, presumably by reducing infection burden. World Health Organization (WHO) issued conditional guidelines for mass azithromycin administration in high-mortality settings in sub-Saharan Africa given concerns for antibiotic resistance. While prolonged twice-yearly MDA has been shown to increase antibiotic resistance in small randomized controlled trials, the objective of this study was to determine if macrolide and non-macrolide resistance in the gut increases with the duration of azithromycin MDA in a larger setting.

**Methods and findings:**

The *Macrolide Oraux pour Réduire les Décès avec un Oeil sur la Résistance* (MORDOR) study was conducted in Niger from December 2014 to June 2020. It was a cluster-randomized trial of azithromycin (A) versus placebo (P) aimed at evaluating childhood mortality. This is a sub-study in the MORDOR trial to track changes in antibiotic resistance after prolonged azithromycin MDA. A total of 594 communities were eligible. Children 1 to 59 months in 163 randomly chosen communities were eligible to receive treatment and included in resistance monitoring. Participants, staff, and investigators were masked to treatment allocation.

At the conclusion of MORDOR Phase I, by design, all communities received an additional year of twice-yearly azithromycin treatments (Phase II). Thus, at the conclusion of Phase II, the treatment history (1 letter per 6-month period) for the participating communities was either (PP-PP-AA) or (AA-AA-AA). In Phase III, participating communities were then re-randomized to receive either another 3 rounds of azithromycin or placebo, thus resulting in 4 treatment histories: Group 1 (AA-AA-AA-AA-A, *N* = 51), Group 2 (PP-PP-AA-AA-A, *N* = 40), Group 3 (AA-AA-AA-PP-P, *N* = 27), and Group 4 (PP-PP-AA-PP-P, *N* = 32).

Rectal swabs from each child (*N* = 5,340) were obtained 6 months after the last treatment. Each child contributed 1 rectal swab and these were pooled at the community level, processed for DNA-seq, and analyzed for genetic resistance determinants. The primary prespecified outcome was macrolide resistance determinants in the gut. Secondary outcomes were resistance to beta-lactams and other antibiotic classes. Communities recently randomized to azithromycin (groups 1 and 2) had significantly more macrolide resistance determinants than those recently randomized to placebo (groups 3 and 4) (fold change 2.18, 95% CI 1.5 to 3.51, *P*_*unadj*_ < 0.001). However, there was no significant increase in macrolide resistance in communities treated 4.5 years (group 1) compared to just the most recent 2.5 years (group 2) (fold change 0.80, 95% CI 0.50 to 1.00, *P*_*adj*_ = 0.010), or between communities that had been treated for 3 years in the past (group 3) versus just 1 year in the past (group 4) (fold change 1.00, 95% CI 0.78 to 2.35, *P*_*adj*_ = 0.52). We also found no significant differences for beta-lactams or other antibiotic classes.

The main limitations of our study were the absence of phenotypic characterization of resistance, no complete placebo arm, and no monitoring outside of Niger limiting generalizability.

**Conclusions:**

In this study, we observed that mass azithromycin distribution for childhood mortality among preschool children in Niger increased macrolide resistance determinants in the gut but that resistance may plateau after 2 to 3 years of treatment. Co-selection to other classes needs to be monitored.

**Trial registration:**

NCT02047981 https://classic.clinicaltrials.gov/ct2/show/NCT02047981.

## Abstract 2

Thuy Doan and co-workers explore the impact of mass azithromycin distribution on the antibiotic resistance in the gut of Nigerien pre-school children.

## Author summary

**Why was this study done?:**

- Mass azithromycin distributions to children aged 1 to 59 months in sub-Saharan countries improve childhood mortality.
- Prolonged mass azithromycin distributions may be necessary until a definitive solution is found for child mortality. However, mass azithromycin distributions also select for antibiotic resistance.
- Here, we compare communities randomized to different treatment histories to assess how antibiotic resistance changes over several years.

**What did the researchers do and find?:**

- MORDOR was a cluster-randomized trial of azithromycin versus placebo aimed at evaluating childhood mortality. Randomization was performed at the village level.
- We conducted a sub-study of the MORDOR trial to track changes to antibiotic resistance in the gut, and 5,340 children from 150 villages were included. Rectal swabs were obtained, pooled, and sequenced for antibiotic resistance.
- We found that macrolide resistance determinants were increased in the gut of children whose villages were treated with twice-yearly azithromycin administration within the past 2 years compared to those who were treated with placebo.
- Longer periods of treatment with azithromycin were not associated with increased macrolide resistance and no changes to non-macrolide resistance determinants were detected.
- The main limitations of this study include the lack of phenotypic resistance assessment, the absence of a treatment arm where communities received a placebo for all 5 years, and that interpretations are limited to the community level in the Dosso region of Niger.

**What do these findings mean?:**

- These findings are consistent with prior smaller studies showing that twice-yearly azithromycin treatment, at the dose recommended to improve childhood mortality, selects for macrolide resistance. However, resistance may plateau after several years, rather than continuing to increase.
- Non-macrolide resistance determinants were not significantly different between treatment arms, although *p*-values and confidence intervals need to be interpreted with caution.
- These findings suggest that careful surveillance for antibiotic resistance should be continued for mass drug distribution for childhood mortality.

## Introduction

Mass antibiotic distribution remains the pillar of multiple public health programs, including those championed by the World Health Organization (WHO). Thus far, over 1 billion doses of azithromycin have been distributed to eliminate trachoma, a blinding disease caused by repeated ocular infection with *Chlamydia trachomatis* [1]. Mass azithromycin distribution for childhood mortality is being considered by various sub-Saharan African countries and WHO [1,2]. Despite proven benefits, the intervention is not without controversy given the emergence of both phenotypic and genetic evidence of antimicrobial resistance [3–6]. Concern is for both an increase resistance to macrolides (including azithromycin and erythromycin), but also co-selection to other classes of antibiotics such as beta-lactams (including penicillin resistance).

Azithromycin for trachoma provides the largest body of evidence for resistance due to mass drug administration (MDA) to date [7]. Communities treated with azithromycin have an increased prevalence of macrolide resistance, which can be phenotypically or genetically detected in *Streptococcus pneumoniae*, *Staphylococcus aureus*, and *Escherichia coli* [8–10]. Reassuringly, resistance prevalence appears to decrease after MDA discontinuation [11]. Recently, due to the ease of transporting DNA coupled with the relative accessibility of high-throughput sequencing and improved bioinformatics, surveillance for resistance in low-resource settings has benefitted from unbiased genetic analysis. This approach has allowed for the detection of all potential antimicrobial resistance determinants in a given sample and has revealed some unexpected results. In Nigerien children, prolonged twice-yearly azithromycin MDA may have led to the co-selection of other classes of antimicrobial resistance determinants in the gut, including beta-lactams and aminoglycosides [12]. Those findings, however, came from a small subset of communities in the MORDOR study [12]. The continuation of the MORDOR mortality study in Niger provided a unique opportunity to evaluate resistance outcomes with azithromycin MDA in a far larger population. Here, we report the findings of the gut resistome of 5,340 Nigerien preschool children from 150 communities randomized to receive up to 9 twice-yearly azithromycin treatments.

## Methods

### Trial design

MORDOR (*Macrolide Oraux pour Réduire les Décès avec un Oeil sur la Résistance*, clinicaltrials.gov # NCT02048007) was a cluster-randomized controlled trial that showed a 14% mortality reduction over 2 years of twice-yearly azithromycin MDA in children aged 1 to 59 months in Niger, Malawi, and Tanzania [13]. It was prespecified that after 2 years, all communities would receive an additional year of azithromycin treatment for all communities (i.e., azithromycin communities would continue to receive 2 more twice-yearly doses of azithromycin and the placebo communities would receive 2 twice-yearly doses of azithromycin; Phase II) [14]. In Phase III, after year 3 (at the conclusion of Phase II), half of the communities were randomized to receive placebo again (**Figure A in** S1 Appendix), as there was concern that after cessation of MDA, mortality might increase to even higher levels than pre-MDA, while levels of resistance remained higher than they would have been without MDA. It was in this large mortality study setting of Phase III that communities were randomly selected for rectal sample collection for macrolide and non-macrolide resistance analysis.

Prior to this sub-study, we monitored for various biomarkers of infection and antibiotic resistance in 30 designated morbidity communities, spanning across the same Dosso region of Niger as the mortality communities in the MORDOR study. The mortality and morbidity studies in MORDOR were parallel trials conducted at the same time. Because childhood mortality is a rare event, the number of mortality communities included in MORDOR was much larger than the number for the morbidity communities (615 communities versus 30 communities). The communities included in this study were themselves part of the large mortality study of MORDOR I, II, and III described above, and thus better reflect the selection for antibiotic resistance where mortality was evaluated as the outcome [3,14]. Of the 163 communities asked, 154 communities agreed to sample collection for this sub-study. The trial started in December 2014 and sample collection for this study concluded in January 2020, although the final survey for MORDOR did not conclude until June 2020.

### Ethics statement

The University of California, San Francisco (UCSF) Committee for Human Research and the Ethical Committee of the Niger Ministry of Health provided ethical oversight. Verbal informed consent was obtained from guardians of eligible children. No incentives were offered. All activities followed the Declaration of Helsinki.

### Study setting and recruitment

The trial was conducted in the districts of Boboye and Loga in Niger. Communities participating in MORDOR Phase I trial were informed of the findings and recruited to continue in the study for Phases II and III.

### Eligibility criteria

Randomization was at the community level, including communities with a population between 200 to 2,000 inhabitants in the previous census. All children within chosen communities who were aged 1 to 59 months weighing at least 3,800 g were eligible for treatment.

### Randomization and masking

In MORDOR I, 646 communities were randomized to azithromycin or placebo MDA. As prespecified, all communities were treated with azithromycin in year 3 because a statistically significant difference in mortality was found [14]. These same communities were re-randomized in Phase III to azithromycin or placebo MDA, without regard to their previous randomization. In this study, 163 communities were randomly selected from MORDOR Phase III (blocked on Phase I assignment, but not on Phase III rerandomization). This randomization scheme reflected a conception of the Phase I comparison as being a central objective. Randomization was generated with the use of R software, version 3.5.1 (R Foundation for Statistical Computing; performed by TCP). While all communities received azithromycin during year 3, participants, field staff, and investigators remained unaware of the original treatment assignment during years 1 to 2 of MORDOR Phases I and III.

### Sample collection

A sample of 40 children (age range 1 to 59 months) in each community was randomly selected for sample collection from the 54-month census. Rectal swabs were then obtained 6 months after the 9th MDA (60-month time point, end of Phase III). Samples were placed in Norgen stool collection kit (Norgen Biotek Corp, Ontario, Canada) to preserve nucleic acids. Samples were stored on ice in the field and at −20°C in Niger until shipment to UCSF for longer-term storage at −80°C until sample processing.

### DNA-sequencing and bioinformatics

All 5,340 collected rectal samples were respectively pooled at the community level, with each pool representing an analog average load of that community. Four communities with less than 10 samples collected were excluded from sequencing [15]. Total DNA was extracted from 150 pooled rectal samples using the Norgen stool DNA isolation kit (Norgen Biotek Corp, Ontario, Canada) per manufacturer’s instructions and prepared for DNA sequencing libraries using the New England Biolabs’ (NEB, Ipswich, Massachusetts, United States of America) NEBNext Ultra II DNA Library Prep Kit and then amplified with 10 PCR cycles and sequenced on the NovaSeq 6000 instrument (Illumina, San Diego, California, USA) using 150-nucleotide (nt) paired-end sequencing. Sequencing reads were analyzed for antibiotic resistance determinants as previously described [16]. Briefly, host reads were removed, and non-host reads were quality-filtered and aligned to the MEGARes reference antimicrobial database (version 2.0.0) using BWA with default settings [17,18]. Each identified antibiotic resistance determinant was grouped at the class level (such as macrolide, aminoglycosides, beta-lactams) using Resistome Analyzer (https://github.com/cdeanj/resistomeanalyzer) and normalized by the total number of non-host reads in the respective pooled sample. Macrolide resistance determinants belong to the macrolide-lincosamide-streptogramin (MLS) class. Those resistance mechanisms include 23S rRNA methyltransferases, macrolide phosphotransferases, macrolide resistance efflux pumps, lincosamide nucleotidyltransferases, macrolide-resistance 23S rRNA mutation, macrolide esterases, steptogramin A O-acetyltransferease, macrolide glycosyltransferases, and steptrogramin B ester bond cleavage.

### Outcomes

The prespecified primary outcome was the normalized macrolide resistance determinants in each of the 4 treatment groups. Prespecified secondary outcomes included resistance to beta-lactams and other antimicrobial classes.

### Trial oversight

A Data and Safety Monitoring Committee (DSMC) consisting of experts in biostatistics, trial design, ethics, epidemiology, and infectious disease oversaw the trial. The DSMC met annually during the course of the trial to review and approve the study design prior to the start of enrollment and to review any proposed changes to the study. Any serious adverse event determined to be possibly related to study participation was reported to the DSMC in real time and to the UCSF Committee for Human Research within 48 h.

### Sample size

We estimated that 160 total communities would provide approximately 80% power to detect a 3.5-fold difference in any pairwise comparison (assuming *P* < 0.01, and an approximate SD of the log base 2 of the read count (plus 1) of 2.25). The SD of the log read count was derived from the placebo arm of the MORDOR 36-month data [12]. This detectable effect size (3.5-fold) was approximately half of what was observed in the MORDOR 36-month results [12].

### Statistical methods

Normalized reads at the antibiotic class-level were used for all analyses. Analyses were performed using a community-level summary statistic, which accounted for the community-level randomization [19].

#### Primary outcome

Pairwise Wilcoxon rank-sum tests for macrolide resistance determinants between each of the treatment history groups, accounting for multiple comparisons by means of the Westfall–Young min-P method [20], which are reported as adjusted *P*-values. All randomized communities were included in the permutation testing, which was based on 100,000 replications. As a methodological sensitivity analysis, the Kruskal–Wallis (nonparametric one-way ANOVA), comparing all 4 arms was used. No adjustments for other covariates were undertaken.

#### Secondary outcomes

For each other drug resistance category (e.g., beta-lactams, aminoglycosides), the community-level normalized read counts for the 4 study arms were compared pairwise using the Wilcoxon rank-sum test. Since this required 6 comparisons for each drug resistance category, we prespecified the use of the Westfall–Young step-down min-P adjustment procedure for each category [20]. We prespecified reporting of min-P findings for macrolide and beta-lactam resistance; all others are pooled and subject to false discovery rate (FDR) adjustment with prespecified FDR of 0.05. Additionally, we report findings contrasting recent administration of placebo (groups 3 and 4) with recent administration of azithromycin (groups 1 and 2) (Wilcoxon rank-sum test, unprespecified analysis). Fold changes were estimated by transforming normalized read counts using the transformation log(*x* + *epsilon*) where *epsilon* = 0.001 (qualitative results are not sensitive to choice of small *epsilon*) and performing analysis using the unadjusted confidence interval from the Wilcoxon rank-sum test. Specifically, the Hodges–Lehmann estimator was computed on the transformed scale, and the inverse transformation was used to yield an approximate ratio. Results were based on 100,000 independent permutations. As a methodological sensitivity analysis, we also conducted an overall Kruskal–Wallis (nonparametric one-way ANOVA) for each drug resistance category. Calculations were conducted using the R statistics package (R Foundation for Statistical Computing, Vienna, Austria), v. 4.3.0 (package NRejections) [21].

## Results

**Fig 1:**
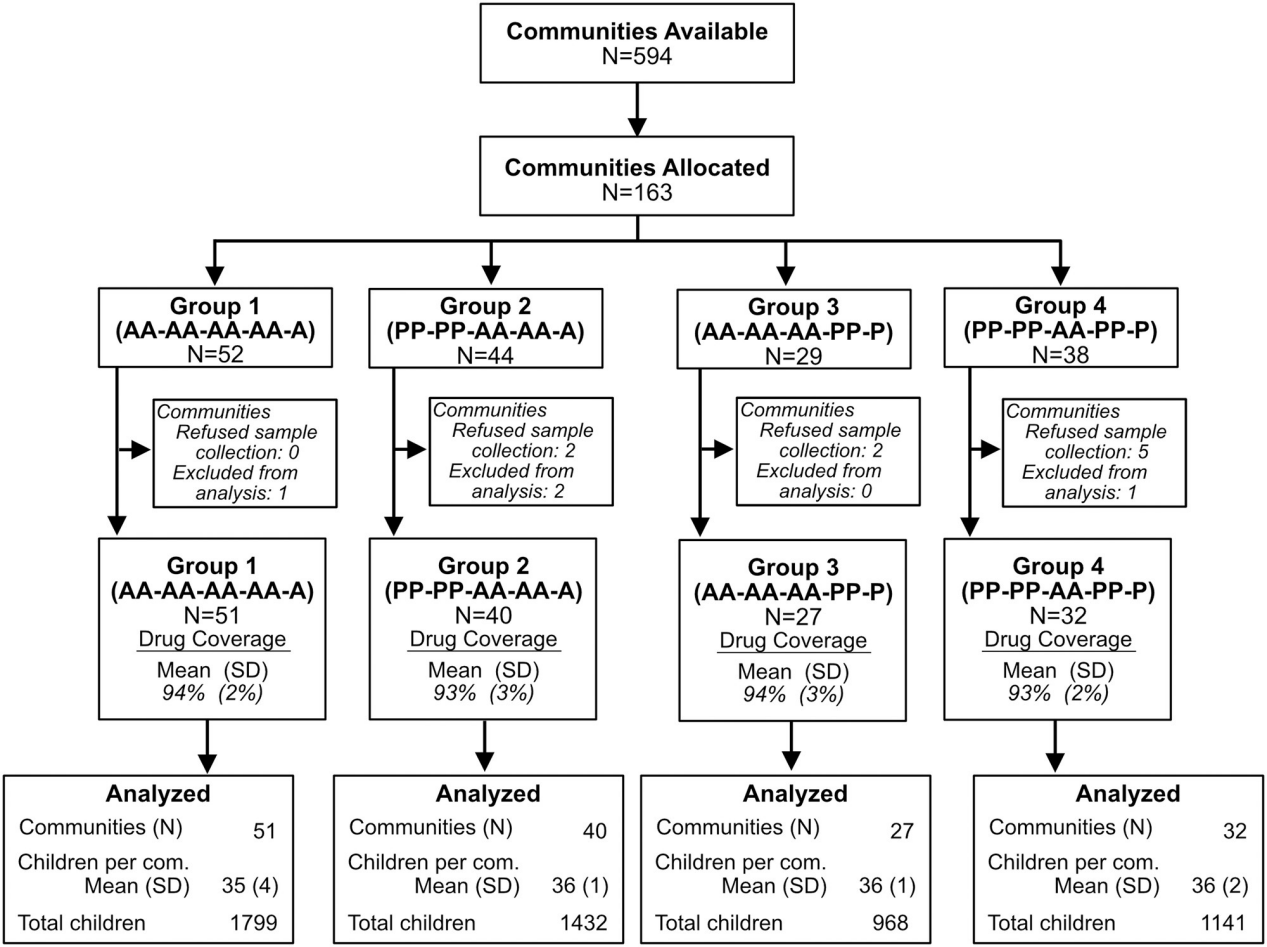
Consort diagram.

As shown in Fig 1, 163 communities from Phase III were randomly selected for potential inclusion with the goal of having 160 communities agreeing to participate. At census, 9 communities refused sample collection, and 4 communities were excluded from sample processing and analysis because fewer than 10 samples were collected. A total of 150 communities remained and contributed to the analysis. Because of the prespecified study design for Phases I, II, and III, the communities fell into 1 of 4 treatment groups: (1) received 9 twice-yearly azithromycin treatments (*N* = 51); (2) received 4 twice-yearly placebo treatments followed by 5 twice-yearly azithromycin treatments (*N* = 40); (3) received 6 twice-yearly azithromycin treatments followed by 3 twice-yearly placebo treatments (*N* = 27); and (4) received 4 twice-yearly placebo, followed by 2 twice-yearly azithromycin, and then followed by 3 more twice-yearly placebo treatments (*N* = 32). The mean (± SD) treatment coverage for the communities in group 1 was 94% (±2%), 93% (±3%) for group 2, 94% (±3%) for group 3, and 93% (±2%) for group 4.

Table 1 summarizes the demographic characteristics of the 5,340 children contributing to sample analysis. Enrolled children across groups had a median age of 30 months (95% CI: 29 months to 32 months). On average, there were 36 children (95% CI: 35 to 37) contributing samples from each community, and 48% (95% CI: 47% to 49%) of children were females.

**Table 1:** Demographic characteristics of communities and children analyzed.

	Group 1	Group 2	Group 3	Group 4
**Treatment**	AA-AA-AA-AA-A	PP-PP-AA-AA-A	AA-AA-AA-PP-P	PP-PP-AA-PP-P
**Number of villages**	51	40	27	32
**Number of children (0–59 mo)**	1,799	1,432	968	1,141
**Mean (±SD) children per village**	35 (±4)	36 (±1)	36 (±1)	36 (±2)
**Age (months), median (IQR)**	30 (23 to 32)	31 (29 to 34)	31 (27 to 33)	29 (26 to 32)
**Female, % (95% CI)**	48% (45% to 51%)	48% (45% to 51%)	47% (43% to 52%)	49% (46% to 52%)

Summary of the demographic characteristics of the 5,340 children contributing to sample analysis. Enrolled children across groups had a median age of 30 months (95% CI: 29 months to 32 months). On average, there were 36 children (95% CI: 35 to 37) contributing samples from each community, and 48% (95% CI: 47% to 49%) of the children were females. All data is computed by children analyzed.

Abbreviations: A, azithromycin; P, placebo; CI, confidence interval; mo, months; SD, standard deviation.

### Primary outcome

**Fig 2:**
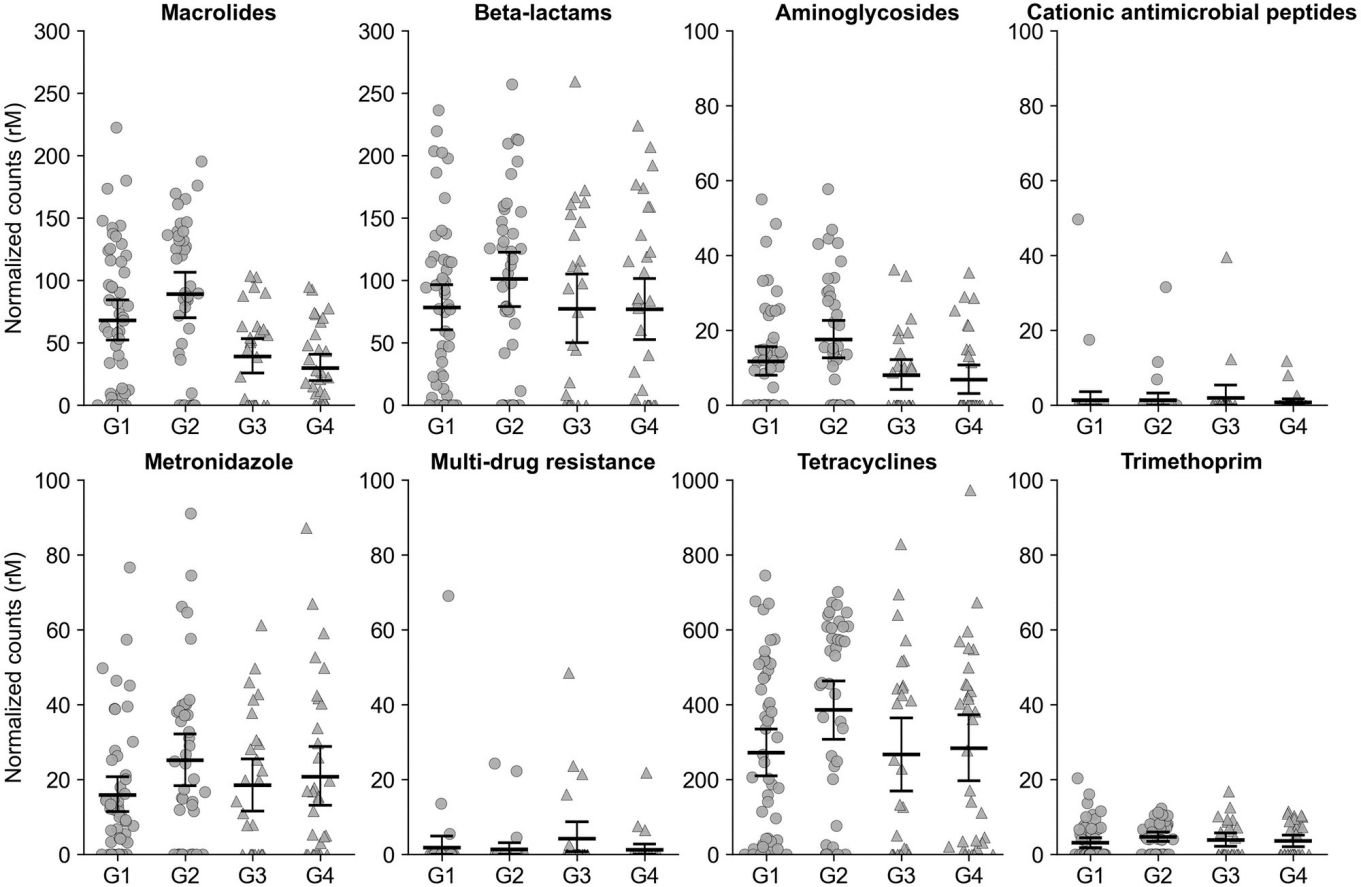
Normalized antibiotic resistance determinants for 4 treatment arms. Bars indicate the mean and 95% confidence intervals calculated by bootstrapping 10,000 times. Each point represents a community. Communities that received twice-yearly azithromycin (circles) and placebo (triangles) during Phase III. Group 1: AA-AA-AA-AA-A; Group 2: PP-PP-AA-AA-A; Group 3: AA-AA-AA-PP-P; Group 4: PP-PP-AA-PP-P. Abbreviations: A, azithromycin; P, placebo; rM, reads per million.

**Fig 3:**
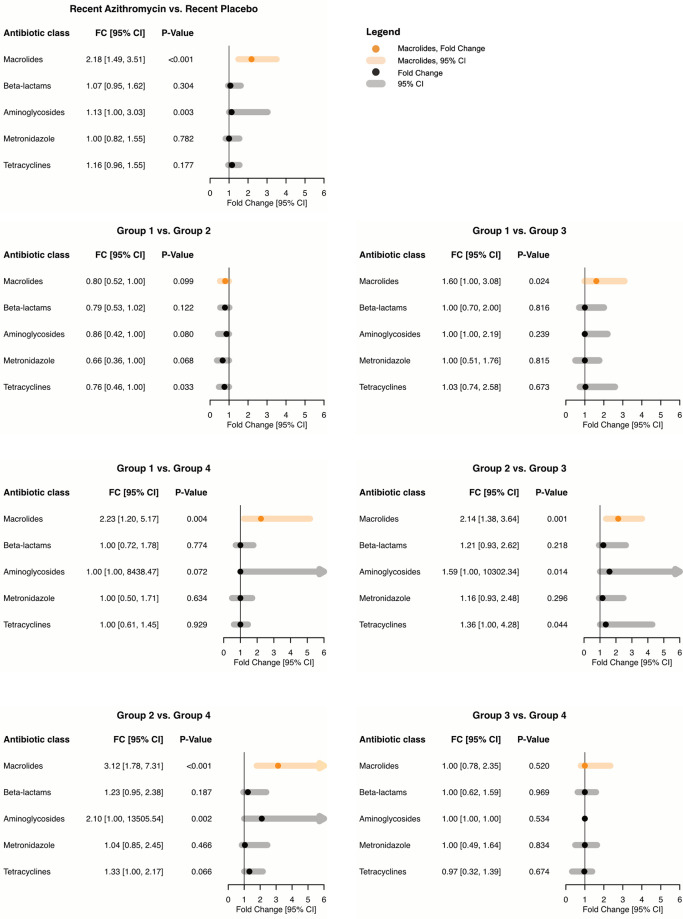
Antibiotic resistance determinants in the gut of children compared among the 4 treatment groups. Top left panel compares recent azithromycin (groups 1 and 2) against recent placebo (groups 3 and 4). Estimated approximate fold change in antibiotic resistance determinants with associated 95% confidence intervals is shown; fold change is an estimate of the factor by which one multiplies the outcome of interest in the reference group to obtain the outcome of interest in the comparison group. Samples were obtained 6 months after the last treatment. Group 1: AA-AA-AA-AA-A; Group 2: PP-PP-AA-AA-A; Group 3: AA-AA-AA-PP-P; Group 4: PP-PP-AA-PP-P. Abbreviations: A, azithromycin; P, placebo; CI, confidence interval.

Fig 2 and **Table A in** S1 Appendix summarize the resulting data structure and statistics. Macrolide resistance determinants in the gut were increased in communities recently receiving twice-yearly azithromycin treatments at the community-level (*P*_*unadj*_ < 0.001, groups 1 and 2 versus groups 3 and 4, fold change 2.18, 95% CI: 1.49 to 3.51; Fig 3, Table 2). In particular, 4.5 years of azithromycin treatments (group 1) resulted in a 2.23-fold (95% CI: 1.20 to 5.17) increase in macrolide resistance determinants compared to 1 year of azithromycin treatment only during year 3 of the study (*P*_*adj*_ = 0.01, Wilcoxon rank-sum (i.e., without covariate adjustment), min-P adjustment). Similarly, communities that did not get treatment during the first 2 years but received azithromycin treatments during the last 3 years of the study (MORDOR II and III) were found to have significantly more macrolide resistance determinants than communities that were randomized to placebo during the last 2 years (MORDOR III) (group 2 versus group 3, 2.14-fold, 95% CI: 1.38 to 3.64, *P*_*adj*_ = 0.003; group 2 versus group 4, 3.12-fold, 95% CI: 1.78 to 7.31, *P*_*adj*_ < 0.001; Wilcoxon rank-sum, min-P adjustment), regardless of prior exposure to azithromycin during the first 2 years of the study (MORDOR I). Finally, we did not find a statistically significant difference in macrolide resistance determinants between 4.5 years of azithromycin treatment (group 1) compared to 2.5 years of treatment (group 2) (0.80-fold, 95% CI: 0.52 to 1.00, *P*_*adj*_ = 0.19, Wilcoxon rank-sum, min-P adjustment) or 3 years of treatment (group 3) (1.60-fold, 95% CI: 1.00 to 3.08, *P*_*adj*_ = 0.06, Wilcoxon rank-sum, min-P adjustment).

**Table 2:** Pairwise Wilcoxon rank-sum test for each antibiotic resistance class detected in the gut of preschool children.

A		Antibiotic classes							
Groups	Treatment	Aminoglycosides	Cationic antimicrobial peptides	Macrolides	Metronidazole	Multi drug resistance	Tetracyclines	Trimethoprim	Beta-lactams
**1 vs. 2**	AA-AA-AA-AA-A vs. PP-PP-AA-AA-A	0.218	0.918	0.187	0.255	0.945	0.140	0.112	0.408
**1 vs. 3**	AA-AA-AA-AA-A vs. AA-AA-AA-PP-P	0.379	0.935	0.063	0.957	0.291	0.908	0.690	0.957
**1 vs. 4**	AA-AA-AA-AA-A vs. PP-PP-AA-PP-P	0.218	0.791	0.014	0.884	0.848	0.927	0.730	0.957
**2 vs. 3**	PP-PP-AA-AA-A vs. AA-AA-AA-PP-P	0.055	0.980	0.003	0.689	0.530	0.157	0.690	0.515
**2 vs. 4**	PP-PP-AA-AA-A vs. PP-PP-AA-PP-P	0.011	0.973	< 0.001	0.840	0.945	0.193	0.611	0.508
**3 vs. 4**	AA-AA-AA-PP-P vs. PP-PP-AA-PP-P	0.533	0.980	0.517	0.957	0.652	0.908	0.927	0.959
**B**									
	Benjamini–Hochberg*	0.066	0.791	NA	0.350	0.350	0.280	0.280	NA

**(A)** Because for each antibiotic outcome, 4 groups imply 6 different pairwise comparisons, min-P adjustment procedure was performed with 100,000 permutations for each antibiotic resistance category. The *P*-values derived from the min-P procedure that were prespecified in the Statistical Analysis Plan are presented in this table. **(B)** Following the analysis within each antibiotic class based on the min-P procedure, a further Benjamini–Hochberg procedure was performed with an FDR prespecified at <0.05 to adjust for multiple antibiotic classes being compared as prespecified, and these values are presented in this table. Abbreviations: A, azithromycin; P, placebo.

*Adjustment of SMALLEST *p*-value for each min-P Wilcoxon.

### Secondary outcomes

There were no detectable changes in the normalized beta-lactam resistance determinants across randomization groups (smallest pairwise *P* ≥ 0.41, Table 2 (A); group 1 versus 2, fold change 0.79, 95% CI: 0.53 to 1.02). We also did not find differences in aminoglycoside resistance determinants when comparing all 4 randomization groups (*P*_*adj*_ = 0.07, Wilcoxon rank-sum, min-P followed by FDR as prespecified). However, the estimated difference was 2.10-fold (95% CI = 1.00 to >100) increase in aminoglycoside resistance determinants in communities randomized to receive azithromycin treatments in the last 3 years of the study (MORDOR II and III, but not MORDOR I, group 2) compared to communities assigned to receive a single year of treatment (MORDOR II, group 4) (*P*_*unadj*_ = 0.01, *P*_*adj*_ = 0.07, Table 2). For the remaining classes of antibiotics (cationic antimicrobial peptides, metronidazole, multi-drug resistance, tetracyclines, and trimethoprim), we were unable to identify a significant difference in genetic resistance determinants between arms (Tables 2 and 3). In methodological sensitivity analysis, FDR-adjusted Kruskal–Wallis tests yielded *P* = 0.047 for differences in aminoglycoside resistance determinants (Table 3).

**Table 3:** Sensitivity analysis. Kruskal–Wallis rank sum test analysis for antibiotic resistance determinants detected in the gut of preschool children.

	Aminoglycosides	Cationic antimicrobial peptides	Macrolides	Metronidazole	Multi drug resistance	Tetracyclines	Trimethoprim	Beta-lactams
**Unadjusted**	0.008	0.823	< 0.001	0.400	0.309	0.093	0.186	0.383
**Adjusted**	0.047	0.823	NA	0.480	0.463	0.280	0.372	NA

A single Kruskal–Wallis test was conducted for each antibiotic outcome (reported as unadjusted; no covariates are possible in this test). The resulting *P*-values were then adjusted with Benjamini–Hochberg procedure for controlling FDR prespecified at <0.05 (excluding macrolides and beta-lactams, according to the prespecified Statistical Analysis Plan).

## Discussion

In this study, we found that azithromycin MDA for childhood mortality selects for macrolide resistance determinants in the gut of preschool children. Here, children in communities randomized to receive azithromycin in the last 1.5 years had more resistance determinants than those randomized to placebo. However, we were unable to find a difference between communities treated for 4.5 years versus just 2.5 years. Thus resistance in the gut may plateau after several MDAs, rather than continue to increase until it saturates the age-group [22]. Previous studies have suggested co-selection of resistance to beta-lactam antibiotics [12,23]. We were unable to confirm this, although we see marginal evidence of an increase in aminoglycoside resistance determinants (as noted in methodological sensitivity analysis). Note that in statistical analysis of adverse outcomes such as resistance reported here, statistical conservatism may de-emphasize potentially important safety indicators. The results of this trial add to the growing body of evidence that supports regular antibiotic resistance surveillance in the setting of azithromycin MDA [24–26].

The short-term beneficial effects of azithromycin MDA for childhood mortality in sub-Saharan and West African countries are clear and may be comparable with other cost-effective interventions [27,28]. In MORDOR Phase I, childhood mortality was reduced by 18% in Niger after 2 years of twice-yearly azithromycin MDA [3]. A similar reduction in childhood mortality was again seen in MORDOR Phase II, when the placebo-treated communities received a year of twice-yearly azithromycin MDA [14]. In Phase II, 1 year of twice-yearly MDA to communities that previously received placebo reduced childhood mortality by 13% in these communities [14]. Antimicrobial resistance monitoring in the morbidity communities showed that there was an increase in the prevalence of macrolide-resistant *S*. *pneumoniae* in the nasopharynx and a selection of macrolide resistance determinants in the gut of children after MORDOR Phase I [8,16]. An additional year of MDA led to an unexpected detection of an increase in genetic resistance determinants for aminoglycosides, beta-lactams, metronidazole, and trimethoprim, suggesting co-selection in the morbidity communities (MORDOR Phase II) [12]. These co-selection effects appeared less robust at the 4-year and 5-year time points, although it was unclear if spillovers through migration and social interactions were diluting the effects as essentially all communities surrounding the 15-placebo communities had received at least 1 year of twice-yearly azithromycin MDA by the 4th and 5th year time points [23,25].

The long-term adverse effects due to the potential spread of antimicrobial resistance with prolonged azithromycin MDA do raise concern. Azithromycin has been distributed for trachoma control for 25 years. WHO treatment guidelines recommend treating entire trachoma-endemic communities annually, resulting in approximately 3 times as many doses as the biannual distributions to preschool children for childhood mortality. Macrolide resistance in trachoma communities returned to a low level soon after cessation of treatment [7,11,29,30]. Furthermore, the results of this study suggest that prior exposure (i.e., during the early MORDOR Phase I study randomization) did not appear to enhance macrolide resistance selection. The increase in macrolide resistance determinants was only detectable between communities that received azithromycin during the last 2 years (MORDOR Phase III) compared to those that received placebo during the last 2 years.

From a surveillance standpoint for azithromycin MDA for either trachoma or childhood mortality, this current study is one of the largest trials attempting to comprehensively characterize antimicrobial resistance in a randomized controlled trial setting [7,24]. While phenotypic analysis may improve the mechanistic understanding at the individual bacteria level or at the species level, the hurdles associated with sample handling in the field, maintaining cold-chains across Niger, and the sheer number of samples and communities involved make such an analysis more difficult. Also, phenotypic analysis typically only analyzes a small number of colonies, while genotypical analysis allows for the potential inclusion of all strains present.

This study had several limitations. First, it lacks an arm where no treatment was provided across 5 years. This was because treatment was prespecified for all participating communities if MORDOR I found a beneficial effect of azithromycin MDA. Thus, all communities had at least 1 year of treatment. Second, as mentioned above, no phenotypic analysis was performed and thus mechanistic information is incomplete. Gut resistome analysis at the community-level allows for the comprehensive cataloging of the potential antimicrobial resistance reservoir. It does not distinguish between whether a single bacterium harbors multiple different antimicrobial resistance genes (either separately or on a single cassette) or if each person in the community carries a different antimicrobial resistance gene in an entirely different bacterial species. Further, because we used normalized read number of resistance determinants in the community, we were unable to differentiate a small number of reads (less relative abundance) in many children versus a large number of reads (high relative abundance) in a single child. These results, however, can guide future surveillance approaches, such as the combination of unbiased resistome surveillance in the gut, *E*. *coli* or *Salmonella* spp in stool, and targeted phenotypic surveillance of *S*. *pneumoniae* in the nasopharynx [26]. Third, the clinical importance of the normalized read number of resistance genetic determinants can itself be difficult to interpret. Future work may elucidate the relationship between genetic determinants and clinically relevant phenotypic resistance in this population. Fourth, randomization was only blocked at baseline, and thus resulted in an imbalance in the number of communities among the treatment groups and a potentially modest loss of power. Fifth, as values derived from a sub-study of a larger trial, the *P*-values and confidence intervals reported in this study should not be interpreted as definitive treatment effects. Moreover, adjusting for familywise error rates increases *P*-values in general, a desirable feature when analyzing exploratory hypotheses or treatment effects across many groups. However, when considering harmful effects in the context of many other analyses, such adjustments reduce our power to find such harmful effects for precisely this reason, an important consideration given that studies are often underpowered to detect rare adverse events. Thus, when considering harmful effects, we prefer to limit the use of such adjustments in the interest of a less statistically conservative or stringent (but more *clinically* conservative) inference. Finally, while large in scope, only Nigerien children were included and thus may limit generalizability in other sub-Saharan African settings where azithromycin MDA may also be used.

In summary, in the Nigerien communities participating in the mortality study of MODROR, we found that azithromycin MDA directly led to a reduction in childhood mortality and an increase in macrolide antibiotic resistance. Longer duration of azithromycin distributions were not associated with increased resistance. Although co-selection of resistance to other classes of antibiotics is possible, these results were not definitive, and only even suggestive with aminoglycosides [31,32]. While azithromycin MDA may not be a long-term solution for childhood mortality, it is currently a feasible and actionable approach to prevent unnecessary deaths in low-resource countries. Indeed, Niger has plans to implement treatment for the entire country and other sub-Saharan countries may follow. Its implementation, however, may need to be coupled with routine and broad surveillance of resistance. Future research should focus on the longer-term effects of azithromycin MDA, spillover effects where the non-target groups (older children and family members) may be at risk, and co-selection of resistance to antibiotics other than macrolides.

## Supporting information

S1 Appendix Supplementary information. (PDF)

S1 CONSORT Checklist CONSORT checklist. (PDF)

## Abbreviations

DSMC: Data and Safety Monitoring Committee
FDR: false discovery rate
MDA: mass drug administration
MLS: macrolide-lincosamide-streptogramin
WHO: World Health Organization
