# Supplementary material for: Prolonged mass azithromycin distributions and macrolide resistance determinants among preschool children in Niger: A sub-study of a cluster-randomized trial (MORDOR)
Source: PLoS Med. 2024 May 6;21(5):e1004386. doi: 10.1371/journal.pmed.1004386 (PMC11073710; doi:10.1371/journal.pmed.1004386)
Supplement: S1 CONSORT Checklist — (PDF) [file pmed.1004386.s002.pdf]

Completed Consolidated Standards of Reporting Trials (CONSORT) checklist with extensions for cluster-randomized and adaptive trials

Section/Topic	Item No	Standard Checklist item	Extension for cluster designs	Extension for adaptive designs	Section of the paper
**Title and abstract**					
	1a	Identification as a randomised trial in the title	Identification as a cluster randomised trial in the title		Title page
	1b	Structured summary of trial design, methods, results, and conclusions (for specific guidance see CONSORT for abstracts)[1,2]	See table below	Structured summary of trial design, methods, results, and conclusions (for specific guidance, see extension of CONSORT for abstracts) [4]	Abstract
**Introduction**					
**Background and objectives**	2a	Scientific background and explanation of rationale	Rationale for using a cluster design		Introduction: Paragraphs 1-2
	2b	Specific objectives or hypotheses	Whether objectives pertain to the cluster level, the individual participant level or both		Introduction: paragraph 2
**Methods**					
**Trial design**	3a	Description of trial design (such as parallel, factorial) including allocation ratio	Definition of cluster and description of how the design features apply to the clusters		Methods: Trial Design and Oversight
	3b			Type of adaptive design used, with details of the pre-planned trial adaptations and the statistical	NA

				information informing the adaptations	
	3c	Important changes to methods after trial commencement (such as eligibility criteria), with reasons		Important changes to the design or methods after trial commencement (such as eligibility criteria) outside the scope of the pre-planned adaptive design features, with reasons	NA
**Participants**	4a	Eligibility criteria for participants	Eligibility criteria for clusters		Methods: Study Setting, Participants, and Eligibility Criteria
	4b	Settings and locations where the data were collected			Methods: Study Setting, Participants, and Eligibility Criteria
**Interventions**	5	The interventions for each group with sufficient details to allow replication, including how and when they were actually administered	Whether interventions pertain to the cluster level, the individual participant level or both		Methods: Intervention
**Outcomes**	6a	Completely defined pre-specified primary and secondary outcome measures, including how and when they were assessed	Whether outcome measures pertain to the cluster level, the individual participant level or both	Completely define pre-specified primary and secondary outcome measures, including how and when they were assessed. Any other outcome measures used to inform pre-planned adaptations	Methods: Outcomes

				should be described with the rationale	
	6b	Any changes to trial outcomes after the trial commenced, with reasons		Any unplanned changes to trial outcomes after the trial commenced, with reasons	NA
**Sample size**	7a	How sample size was determined	Method of calculation, number of clusters(s) (and whether equal or unequal cluster sizes are assumed), cluster size, a coefficient of intracluster correlation (ICC or *k*), and an indication of its uncertainty	How sample size and operating characteristics were determined	Methods: Sample Size
	7b	When applicable, explanation of any interim analyses and stopping guidelines		Pre-planned interim decision-making criteria to guide the trial adaptation process; whether decision-making criteria were binding or non-binding; pre-planned and actual timing and frequency of interim data looks to inform trial adaptations	Methods: Statistical Analysis
**Randomisation:**					
**Sequence generation**	8a	Method used to generate the random allocation sequence			Methods: Randomization and Masking
	8b	Type of randomisation; details of any restriction (such as blocking and block size)	Details of stratification or matching if used	Type of randomisation; details of any restriction (such as blocking and block size); any changes to the allocation rule after trial adaptation decisions; any pre-	Methods: Randomization and Masking

				planned allocation rule or algorithm to update randomisation with timing and frequency of updates	
**Allocation concealment mechanism**	9	Mechanism used to implement the random allocation sequence (such as sequentially numbered containers), describing any steps taken to conceal the sequence until interventions were assigned	Specification that allocation was based on clusters rather than individuals and whether allocation concealment (if any) was at the cluster level, the individual participant level or both		Methods: Randomization and Masking
**Implementation**	10	Who generated the random allocation sequence, who enrolled participants, and who assigned participants to interventions	Replace by 10a, 10b and 10c		Methods: Randomization and Masking
	10a		Who generated the random allocation sequence, who enrolled clusters, and who assigned clusters to interventions		Methods: Randomization and Masking
	10b		Mechanism by which individual participants were included in clusters for the purposes of the trial (such as complete enumeration, random sampling)		Methods: Randomization and Masking
	10c		From whom consent was sought (representatives of the cluster, or individual cluster members, or		Methods: Study Setting, Participants, and Eligibility

			both), and whether consent was sought before or after randomisation		Criteria
**Blinding**	11a	If done, who was blinded after assignment to interventions (for example, participants, care providers, those assessing outcomes) and how			Methods: Randomization and Masking
	11b	If relevant, description of the similarity of interventions			Methods: Randomization and Masking
	11c			Measures to safeguard the confidentiality of interim information and minimise potential operational bias during the trial	Methods: Randomization and Masking
**Statistical methods**	12a	Statistical methods used to compare groups for primary and secondary outcomes	How clustering was taken into account	Statistical methods used to compare groups for primary and secondary outcomes, and any other outcomes used to make pre-planned adaptations	Methods: Statistical Analysis
	12b			For the implemented adaptive design features, statistical methods used to estimate treatment effects for key endpoints and to make inferences	NA

	12c	Methods for additional analyses, such as subgroup analyses and adjusted analyses			Methods: Statistical Analysis
**Results**					
**Participant flow (a diagram is strongly recommended)**	13a	For each group, the numbers of participants who were randomly assigned, received intended treatment, and were analysed for the primary outcome	For each group, the numbers of clusters that were randomly assigned, received intended treatment, and were analysed for the primary outcome	For each group, the numbers of participants who were randomly assigned, received intended treatment, and were analysed for the primary outcome and any other outcomes used to inform pre-planned adaptations, if applicable	Results: Paragraph 1, Figure 1, Figure S1
	13b	For each group, losses and exclusions after randomisation, together with reasons	For each group, losses and exclusions for both clusters and individual cluster members		Results: Participant Enrollment, Baseline Characteristics, Treatment Coverage, Table 1, Figure S1
**Recruitment**	14a	Dates defining the periods of recruitment and follow-up		Dates defining the periods of recruitment and follow-up, for each group	Results: Participant Enrollment, Baseline Characteristics, Treatment Coverage, Figure 1
	14b	Why the trial ended or was stopped		Why the trial or certain treatment arm(s) were stopped outside the scope of pre-planned adaptations	NA
	14c*			Specify what trial adaptation decisions were made in light of	NA

				the pre-planned decision-making criteria and observed accrued data	
**Baseline data**	15	A table showing baseline demographic and clinical characteristics for each group	Baseline characteristics for the individual and cluster levels as applicable for each group	A table showing baseline demographic and clinical characteristics for each group. In presence of marked differences in numbers of randomized participants and those included in interim or final analysis, authors encouraged to report baseline summaries by treatment group for these two populations.	Table 1
	15b*			Summary of data to enable the assessment of similarity in the trial population between interim stages	NA
**Numbers analysed**	16	For each group, number of participants (denominator) included in each analysis and whether the analysis was by original assigned groups	For each group, number of clusters included in each analysis	The number of participants by treatment group should be reported for each analysis at both the interim analyses and final analysis whenever a comparative assessment is performed	Results: Participant Enrollment, Baseline Characteristics, Treatment Coverage, Table 1, Figure S1
**Outcomes and estimation**	17a	For each primary and secondary outcome, results for each group, and the estimated effect size and its precision (such as 95% confidence interval)	Results at the individual or cluster level as applicable and a coefficient of intracluster correlation (ICC or k) for each primary outcome	In ADs, such as population (or biomarker or patient) enrichment, reporting of results as detailed in the standard CONSORT should mirror hypotheses of interest	Results: Primary outcomes, Secondary outcomes, Figure 2, Table 2, Table 3

	17b	For binary outcomes, presentation of both absolute and relative effect sizes is recommended			NA
	17c			Report interim results used to inform interim decision-making	NA
**Ancillary analyses**	18	Results of any other analyses performed, including subgroup analyses and adjusted analyses, distinguishing pre-specified from exploratory			NA
**Harms**	19	All important harms or unintended effects in each group (for specific guidance see CONSORT for harms[3])			Results: NA
**Discussion**					
**Limitations**	20	Trial limitations, addressing sources of potential bias, imprecision, and, if relevant, multiplicity of analyses		Additional considerations include discussion on the implications of deviations from the pre-planned adaptations, interim analysis, protocol amendments on the trial adaptation and results, potential sources of bias introduced by interim analyses or decision-making, Potential heterogeneity in patient characteristics and treatment effects between stages,	Discussion: Limitations

				Potential heterogeneity in patient characteristics and treatment effects between stages, Whether outcome data were sufficient to robustly inform trial adaptations at interim analyses and using adaptation outcome(s) different from the primary outcome(s)	
**Generalisability**	21	Generalisability (external validity, applicability) of the trial findings	Generalisability to clusters and/or individual participants (as relevant)	Additional considerations that may influence the generalisability of results from AD randomised trials	Discussion: Limitations
**Interpretation**	22	Interpretation consistent with results, balancing benefits and harms, and considering other relevant evidence			Discussion: paragraphs 1-4
**Other information**					
**Registration**	23	Registration number and name of trial registry			Abstract, Methods: Trial Design and Oversight
**Protocol**	24	Where the full trial protocol can be accessed, if available			Methods: Trial Design and Oversight
	24b			Where the full statistical analysis plan and other relevant trial documents can be accessed	Methods: Trial Design and Oversight

**Funding**	25	Sources of funding and other support (such as supply of drugs), role of funders		Funding

Extension of CONSORT for abstracts[1,2,4] to reports of cluster randomised trials

Item	Standard Checklist item	Extension for cluster trials	Extension for adaptive trials
**Title**	Identification of study as randomised	Identification of study as cluster randomised	
**Trial design**	Description of the trial design (e.g. parallel, cluster, non-inferiority)		Description of the trial design (eg, parallel, cluster, non-inferiority); include the word "adaptive" in the content or at least as a keyword
**Methods**			
**Participants**	Eligibility criteria for participants and the settings where the data were collected	Eligibility criteria for clusters	
**Interventions**	Interventions intended for each group		
**Objective**	Specific objective or hypothesis	Whether objective or hypothesis pertains to the cluster level, the individual participant level or both	
**Outcome**	Clearly defined primary outcome for this report	Whether the primary outcome pertains to the cluster level, the individual participant level or both	Describe adaptation outcomes similar to the description of pre-specified primary and secondary outcomes. Provide a clinical rationale supporting the use of an adaptation outcome that is different to the primary outcome in order to aid the clinical interpretation of results.
**Randomization**	How participants were allocated to interventions	How clusters were allocated to interventions	
**Blinding (masking)**	Whether or not participants, care givers, and those assessing the outcomes were blinded to group assignment		
**Results**			
**Numbers randomized**	Number of participants randomized to each group	Number of clusters randomized to each group	

**Recruitment**	Trial status[1]		
**Numbers analysed**	Number of participants analysed in each group	Number of clusters analysed in each group	
**Outcome**	For the primary outcome, a result for each group and the estimated effect size and its precision	Results at the cluster or individual participant level as applicable for each primary outcome	
**Harms**	Important adverse events or side effects		
**Adaption decisions made**			Specify what trial adaptation decisions were made in light of the pre-planned decision-making criteria and observed accrued data.
**Conclusions**	General interpretation of the results		
**Trial registration**	Registration number and name of trial register		
**Funding**	Source of funding		

REFERENCES

1 Hopewell S, Clarke M, Moher D, Wager E, Middleton P, Altman DG, et al. CONSORT for reporting randomised trials in journal and conference abstracts. *Lancet* 2008, 371:281-283

2 Hopewell S, Clarke M, Moher D, Wager E, Middleton P, Altman DG at al (2008) CONSORT for reporting randomized controlled trials in journal and conference abstracts: explanation and elaboration. *PLoS Med* 5(1): e20

3 Ioannidis JP, Evans SJ, Gotzsche PC, O'Neill RT, Altman DG, Schulz K, Moher D. Better reporting of harms in randomized trials: an extension of the CONSORT statement. *Ann Intern Med* 2004; 141(10):781-788.

4 Dimairo M, Pallmann P, Wason J, Todd S, Jaki T, Julious S A et al. The Adaptive designs CONSORT Extension (ACE) statement: a checklist with explanation and elaboration guideline for reporting randomised trials that use an adaptive design *BMJ* 2020; 369 :m115 doi:10.1136/bmj.m115
